# Podoconiosis patients’ willingness to pay for treatment services in Northwest Ethiopia: potential for cost recovery

**DOI:** 10.1186/1471-2458-14-259

**Published:** 2014-03-19

**Authors:** Abreham Tamiru, Girmay Tsegay, Moges Wubie, Molla Gedefaw, Sara Tomczyk, Fasil Tekola-Ayele

**Affiliations:** 1 School of Public Health, College of Medicine and Health Sciences, Debre Markos University, P.O. Box 269, Debre Markos, Ethiopia; 2 GAMBY Medical Sciences College, P.O. Box 209, Bahir Dar, Ethiopia; 3 Institute of Tropical Medicine, Antwerp, Belgium; 4 Center for Research on Genomics and Global Health, National Human Genome Research Institute, National Institutes of Health, 20892 Bethesda, MD, USA

**Keywords:** Willingness to pay (WTP), Podoconiosis, Neglected tropical disease, Health policy, Global health, Treatment, Contingent valuation method, Ethiopia

## Abstract

**Background:**

Podoconiosis is non-filarial elephantiasis of the lower legs. It is more commonly found in tropical Africa, Central and South America, and northwest India. In Ethiopia, a few non-governmental organizations provide free treatment to podoconiosis patients, but sustainability of free treatment and scale-up of services to reach the huge unmet need is challenged by resource limitations. We aimed to determine podoconiosis patient’s willingness to pay (WTP) for a treatment package (composed of deep cleaning of limbs with diluted antiseptic solution, soap, and water, bandaging, application of emollient on the skin, and provision of shoes), and factors associated with WTP in northwestern Ethiopia.

**Methods:**

A cross-sectional study was conducted among randomly selected untreated podoconiosis patients (n = 393) in Baso Liben *woreda*, northwestern Ethiopia. The contingent valuation method was used with a pre-tested interviewer-administered questionnaire.

**Results:**

The majority of podoconiosis patients (72.8%) were willing to pay for treatment services. The median WTP amount was 64 *Birr* (US$ 3.28) per person per year. More than one-third of patients (36.7%) were willing to pay at least half of the full treatment cost and 76.2% were willing to pay at least half of the cost of shoes. A multivariate analysis showed that having a higher monthly income, being a woman, older age, being aware of the role of shoes to prevent podoconiosis, and possession of a functional radio were significantly associated with higher odds of WTP.

**Conclusions:**

The considerable WTP estimates showed that podoconiosis treatment could improve sustainability and service utilization. A subsidized cost recovery scheme could reduce treatment costs and more feasibility integrate podoconiosis treatment service with other NTDs and the government’s primary health care system.

## Background

Podoconiosis is a geo-chemical neglected tropical disease (NTD) affecting genetically susceptible people that have prolonged barefoot exposure to red clay soil. It leads to elephantiasis or swelling of the lower legs [1-4]. Podoconiosis is common in more than ten countries across tropical Africa, Central and South America and northwest India [1,5,6]. In endemic areas, podoconiosis-affected patients and families suffer from extreme social stigmatization, physical disability, and economic impairment [7-9].

At the early clinical stages, podoconiosis can be treated using simple and effective measures: regular washing of feet with soap and water, application of emollient on the skin, elevation of the leg at night, compression therapy with elastic bandages, and regular use of footwear [10]. However, podoconiosis has been given little attention by health policy makers and implementing health institutions in endemic countries. For example, in Ethiopia, approximately one million patients are affected by podoconiosis, but only four percent have access to treatment services. These treatment services are provided by non-governmental non-profit organizations [11]. In northern Ethiopia, most of the prevention and treatment services are provided nearly free of charge by the International Orthodox Christian Charities (IOCC) Podoconiosis Treatment and Prevention Project at six treatment clinics [12]. During the past three years, the demand for the treatment has increased and surpassed IOCC’s resources. As a consequence, a large number of podoconiosis patients are on a waiting list. In western and southern Ethiopia, other donor-driven podoconiosis treatment programs have also faced financial challenges that have limited the sustainable provision of treatment and scale-up of services to meet the unmet needs [11].

Treatment cost-recovery options may add to the sustainability of treatment services and allow the integration of podoconiosis treatment in the primary health care system of government health facilities. In the present study, we determined the willingness to pay (WTP) of podoconiosis patients in northern Ethiopia to understand whether cost-recovery can be proposed as an option for sustainable treatment and control of podoconiosis.

## Methods

### Study area

The study was conducted in Baso Liben *woreda* (a government administrative region in Ethiopia, equivalent to a district), one of the 20 *woreda*s in East Gojam Zone, northwestern Ethiopia. Its main town has an elevation of 2,211 meters above sea level. Baso Liben *woreda*’s projected population size in the year 2013 was estimated to be 152,598 people, of which 95% live in rural areas and depend on subsistence farming for a living [13].

### Study design and sampling

This was a cross-sectional institution-based study. The source of the study participants was podoconiosis patients in Baso Liben *woreda* on the waiting list of IOCC’s Podoconiosis Treatment and Prevention Project (n = 3,800). Using sample size calculation for a single population proportion and assuming a 95% CI (z = 1.96) for the margin of error to be no wider than 5%, and a maximum single proportion (50%), the minimum sample size becomes 384 individuals. We added 9 individuals on top of this for contingency. Adult podoconiosis patients were selected from the waiting list using the simple random sampling technique. First, the names of individuals on the waiting list at the IOCC Baso Liben treatment site were written on pieces of papers. Next, the pieces of papers were folded and mixed, and 393 study participants were randomly chosen to be included in the study.

### Data collection tool and procedures

Data were collected using a structured questionnaire that had two parts. Part one included questions that assessed the demographic, clinical, shoe wearing, and socio-economic characteristics of the study participants. Part two assessed the study participants’ WTP for podoconiosis treatment services. To assess WTP, the contingent valuation method (CVM) was used. CVM is a questionnaire-based method used to elicit the monetary value a person is willing to pay for a health care service [14]. First, a detailed explanation was given to all participants about the components of the treatment services provided by IOCC’s Podoconiosis Treatment and Prevention Project, frequency of visits and schedules, types of health workers involved, and infrastructure of health facilities available for the treatment services. The treatment components include deep cleaning of limbs with diluted antiseptic solution, soap, and water, bandaging, application of emollient on the skin, and provision of shoes. Next, the respondents were asked a ‘yes’ or ‘no’ question about whether or not they would agree to pay for these treatment services. If the response was ‘no’, the respondents were asked the reason. If the response was ‘yes’, the respondents were asked an open-ended contingent valuation question about the maximum amount that they would be willing to pay for the treatment services per person per year. Following this open-ended question, every study participant was asked a series of binary (i.e. dichotomous choice) format questions based on the ‘bidding game’ method [14]. In the ‘bidding game’ method, respondents are asked whether or not they would be willing to pay a given amount and they are then asked follow-up questions about higher or lower amounts [14]. In this study, the series of questions was: (a) Are you willing to pay the full IOCC cost of treatment for podoconiosis (330 *Birr* or US$ 16.92 per person per year)? (if no, go to b); (b) Are you willing to pay 3/4^th^ of the full cost (248 *Birr* or US$ 12.72 per person per year)? (if no, go to c); (c) Are you willing to pay half of the full cost (165 *Birr* or US$ 8.46 per person per year)? Shoes are the main component of podoconiosis treatment and prevention. Therefore, affected individuals were asked similar questions about WTP for shoes given other services are provided for free starting with the IOCC cost of shoe production for one person (250 *Birr* or US$ 12.82). Eight nurses administered the questionnaires, and two public health officers supervised the data collection. Data collectors and supervisors were trained by the lead author of this paper (AT) for three days. Topics covered during the training included objectives of the study, interviewing techniques, and practical exercises. Pilot testing of the questionnaire was done before in a neighboring village the actual data collection. Data were collected from May to June 2013.

### Data analysis

The raw data were entered and cleaned in EPI INFO statistics program version 6.04, and analyzed using the Statistical Package Social Sciences (SPSS) statistics program version 16.0. Descriptive statistics was done using summary statistics such as frequencies, average estimates (i.e., mean or median), and summary figures. ANOVA, univariate, and backward stepwise multivariate logistic regression analyses were performed to model factors associated with participants’ willingness to pay. The level of significance was set to be 0.05.

### Ethical considerations

Ethical approval for the study was obtained from Debre Markos University in northern Ethiopia. Support letters were obtained from the regional and zonal health administrations. Informed consent was obtained from all study participants.

## Results

### Socio-demographic and clinical characteristics of the study participants

A total of 393 podoconiosis patients participated in the study (100% response rate). The mean age of the study participants was 40.5 years (standard deviation [s.d.] = 13.5), 40.7% of the study participants were women, 69.5% were married, 86.5% were farmers, and 82.7% could not read and write. The mean family size per household was 5.2 individuals (s.d. = 2.6), and the majority (64.1%) of the study participants perceived their household’s socio-economic status to be lower than the village average. Based on a clinical staging system for podoconiosis [15], 267 (67.9%) participants had early clinical disease (i.e. stage I or II), and 126 (32.1%) had advanced clinical disease (i.e., stage III, IV, or V). A total of 78 (19.8%) study participants said that they had at least one podoconiosis-affected family member. Half of the study participants (55% of women and 45.5% of men) were barefoot during the interview, and 135 study participants (34.4%; 38.1% of women and 31.8% of men) said that they had never worn shoes (Table 1).

**Table 1:** Characteristics of the study participants (n = 393)

**Characteristics**		**Number (%)**
Sex	Male	233 (59.3)
	Female	160 (40.7)
Age in years (mean, SD)		40.5 (SD = 13.5)
Occupation	Farmer	340 (86.5)
	Housewife	17 (4.3)
	Private business	22 (5.6)
	Other	14 (3.6)
Education	Can read and write	68 (17.3)
	Cannot read and write	325 (82.7)
Marital status	Married	273 (69.5)
	Divorced	53 (13.5)
	Widowed	34 (8.7)
	Single	33 (8.4)
Monthly household income in Birr* (n = 365), median (range)	100-180	85 (23.3)
	181-300	92 (25.2)
	301-500	104 (28.5)
	501-2500	84 (23.0)
	300 (50–2502)	
Patients’ comparison of their household socio-economic status with others in the same village	Below average	252 (64.1)
	Average	129 (32.8)
	Above average	12 (3.1)
Clinical stage	Stage I	28 (7.1)
	Stage II	239 (60.8)
	Stage III	121 (30.8)
	Stage IV	4 (1.0)
	Stage V	1 (0.3)
Have other podoconiosis-affected family member(s)	Yes	78 (19.8)

*Birr is the unit of currency in Ethiopia.

### Perceptions of the study participants about podoconiosis

The majority (65.7%) of the study participants believed that podoconiosis is caused by evil spirits or did not know the cause of podoconiosis. Only 34 (8.6%) study participants mentioned that red clay soil has a causal association with the development of podoconiosis; the remaining 91.4% had misconception about the cause of podoconiosis. A total of 332 (84.5%) study participants said that podoconiosis is preventable. Of these, most perceived that podoconiosis can be prevented by avoiding marriage with members of podoconiosis-affected families (48.8%) or physical contact with podoconiosis affected individuals (38%). A total of 94 (28.3%) and 92 (27.7%) study participants mentioned that podoconiosis can be prevented by wearing shoes and washing feet with soap and water, respectively (Table 2).

**Table 2:** Perceptions and experiences about podoconiosis prevention

**Question**	**Response**	**Number (%)**
What is the cause of podoconiosis?	Evil Spirit	178 (45.3)
	Insect	15 (3.8)
	Soil/mud	34 (8.6)
	Contact with dirty water	6 (1.5)
	Do not know	60 (20.4)
Is podoconiosis preventable?	Yes	332 (84.5)
	No	61 (15.5)
If the response to the above question is ‘Yes’: How can one prevent podoconiosis?	Wearing shoes	94 (28.3)
	Washing foot with soap and water	92 (27.7)
	Avoiding marriage with podoconiosis-affected family members	162 (48.8)
	Avoiding contact with podoconiosis patients	126 (38.0)

### Willingness to pay for podoconiosis treatment services

**Figure 1:**
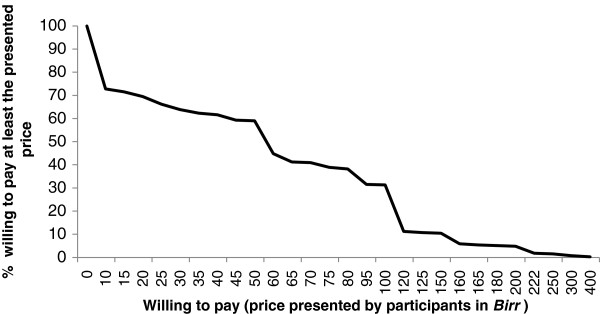
Demand curve for maximum willingness to pay for podoconiosis treatment services.

A total of 286 (72.8%) study participants were willing to pay for podoconiosis treatment services. The study participants who chose not to pay (n = 107; 27.2%) said that they could not afford to pay (93.4%) or had low confidence in the effectiveness of the treatment (6.6%). The mean household monthly income of study participants who were not willing to pay was lower than that of participants who were willing to pay (212.2 *Birr* vs. 455.65 *Birr*; t = −10.39, p < 0.0001). Through the open-ended WTP question, we found that the median amount a person was willing to pay for the treatment services was 64 *Birr* (US$ 3.28; range: 0–400 *Birr* or US$ 0–20.51) per person per year. The demand for treatment services declined as price increased. This decline was slow between 10 *Birr* (US$ 0.51) and 50 *Birr* (US$ 2.56) after which it fell quickly. For example, 59% of the study participants had a WTP of at least 50 *Birr* (US$ 2.56), whereas only 31.1% had a WTP of at least 100 *Birr* (US$ 5.13) (Figure 1). Over half of the individuals were willing to pay for other affected family members (Table 3).

**Table 3:** Willingness to pay for podoconiosis treatment services

**Willingness to pay characteristics**		**Number (%)**	**Cumulative number (%)**
Willing to pay for podoconiosis treatment services (n = 393)?	Yes	286 (72.8)	NA
	No	107 (27.2)	NA
Of those that agreed to pay (n = 286), individuals willing to pay the following:	Full cost (330 Birr*)	44 (15.4)	44 (15.4)
	3/4^th^ of full cost (248 *Birr*)	14 (4.9)	58 (20.3)
	½ of full cost (165 *Birr*)	47 (16.4)	105 (36.7)
Willing to pay for shoes if they get treatment supply free (n = 286)	Full cost (250 *Birr*)	88 (30.8)	88 (30.8)
	3/4^th^ of full cost (188 *Birr*)	24 (8.4)	112 (39.2)
	½ of full cost (125 *Birr*)	106 (37.1)	218 (76.3)
Would you be willing to pay for other family members (n = 393)?	Yes	218 (55.5)	NA
	No	175 (44.5)	NA

*Birr is the unit of currency in Ethiopia.

NA = not applicable.

Among the study participants who agreed to pay (n = 286), some agreed to pay the full amount (n = 44; 15.4%), three-fourths the amount (n = 14; 4.9%), or half the amount (n = 47; 16.4%) of the current IOCC average treatment cost of 330 *Birr* (US$ 16.92) per person per year. The cumulative proportions showed that 44 (15.4%), 58 (20.3%), and 105 (36.7%) study participants were willing to pay 100%, at least 75%, and at least 50% of the average treatment cost of IOCC. When asked how much they would be willing to pay for shoes if other treatment supplies were free, some study participants were willing to pay the full amount (n = 88; 30.8%), three-fourths the amount (n = 24; 8.4%) or half the amount (n = 106; 37.1%) of the current IOCC shoe production cost of 250 *Birr* (US$ 12.82) for one person. The cumulative proportions showed that 88 (30.8%), 112 (39.2%), and 218 (76.2%) study participants were willing to pay 100%, at least 75%, and at least 50% of the shoe production cost if other treatment services are provided for free (Table 3).

### Factors associated with willingness to pay for podoconiosis treatment services

**Figure 2:**
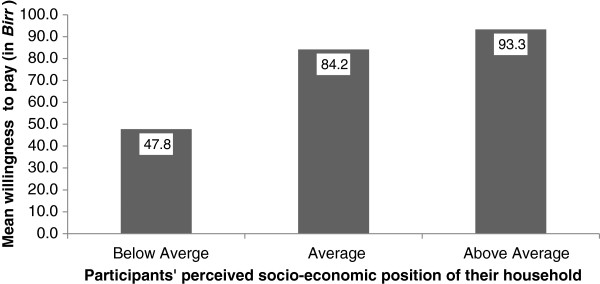
Perceived household socio-economic status and mean willingness to pay for podoconiosis treatment services.

Study participants who perceived their households to be poorer than the village average were willing to pay about half as much as those who considered their socioeconomic position average or better (ANOVA F = 36.5, p < 0.0001) (Figure 2). The multivariate analysis showed that being a woman, older age, having knowledge that podoconiosis can be prevented by wearing shoes, possession of a functional radio, higher income, recent history of paying for treatment services for other conditions, and production of wheat were associated with increased WTP for podoconiosis treatment services (Table 4). There was no statistically significant difference in WTP between patients with early and advanced clinical disease stages.

**Table 4:** Factors associated with willingness to pay for podoconiosis treatment services

**Variable**		**Willing to pay**		**Crude OR**	Adjusted OR^*^
		**Yes**	**No**	**(95% CI)**	**(95% CI)**
Sex	Male	175	58	1.33 (0.85,2.08)	0.43 (0.19,0.96)¥
	Female	111	59	1	1
Educated	No	236	89	0.96 (0.53,1.72)	0.44 (0.23,0.56)
	Yes	50	18	1	1
Age in years	18-30	85	31	0.69 (0.35,1.34)	0.36 (0.13,0.51)¥
	31-38	56	26	0.54 (0.27,1.09)	0.21 (0.07,0.64)¥
	39-50	77	33	0.58 (0.29,1.14)	0.36 (0.16,0.71)¥
	51-80	68	17	1	1
Monthly income in *Birr*	100-180	37	48	0.05 (0.02,0.13)	0.16 (0.05,0.50)¥
	181-300	57	35	0.10 (0.04,0.28)	0.42 (0.13,0.74)¥
	301-500	97	7	0.88 (0.27,2.87)	0.79 (0.63,0.92)¥
	501-2500	79	5	1	1
Believed that podoconiosis is preventable by wearing shoes	Yes	249	83	1.95 (1.16,3.44)	4.08 (3.45,5.54)¥
	No	37	24	1	1
Paid for treatment during the past month	Yes	59	14	1.27 (0.92,3.24)	3.45 (1.27,9.34)¥
	No	227	93	1	1
Family possessed a functional radio	Yes	97	12	4.06 (2.12,7.77)	4.87 (1.89,12.55)¥
	No	189	95	1	1
Family harvested wheat	Yes	231	54	4.12 (2.55,6.66)	3.79 (1.77,8.11)¥
	No	55	55	1	1
Wearing shoes during the interview	Yes	204	54	2.44 (1.55,3.85)	2.44 (1.22,4.9)¥
	No	82	53	1	1

OR = odds ratio, CI = confidence interval, Ref = reference.

*Adjustment was made for: sex, education, age, monthly income, belief on preventability of podoconiosis, previous history of paying for treatment service, possession of goat, radio, and donkey, wheat harvesting, wearing shoes during interview.

¥P-value is <0.05.

## Discussion

In this first study to assess WTP for podoconiosis treatment services, we found that the majority (72.8%) of the study participants in Baso Liben *woreda* were willing to pay. The proportion of individuals willing to pay for podoconiosis treatment was greater than the proportion of individuals willing to pay for injectable contraceptives in Tigray, northern Ethiopia (68%) [16], but lower than the proportion willing to pay for insecticide-treated bed nets in Arbaminch, southern Ethiopia (86%) [17], and lipid-based nutrient supplements for young children in four urban sites of Ethiopia (96%) [18]. These differences may be due to differences in socio-economic and demographic characteristics of the study populations. Individuals may also perceive malaria and malnutrition more severe with fatal consequences as compared to podoconiosis which is disfiguring, physically disabling, and psychologically and economically traumatizing but not fatal [6]. Despite these differences, the WTP for podoconiosis reported in this study is considerably high given the fact that podoconiosis is a neglected tropical disease that affects the poor, and the minimal level of awareness and wide misconceptions about podoconiosis in our study sample (91.4%) and in a wider study population in northern Ethiopia [19,20].

The proportion of respondents willing to pay the full price doubled from 15.4% to 36.7% when the fee requested dropped by half. Furthermore, the proportion of respondents willing to pay doubled from 36.7% that were willing to pay at least half IOCC’s cost of full treatment package to 76.2% that were willing to pay at least half IOCC’s cost of shoes when other treatment services were given for free. The non-shoe component of podoconiosis treatment accounts 24% (80 *Birr*) of IOCC’s total treatment cost. Although full cost recovery or full user fee programs would not be feasible, we have found that incentives such as free service provision, however small, influence podoconiosis patients’ WTP for treatment and utilization of the overall treatment package may be increased. This indicates that partial cost recovery programs could be introduced to increase coverage of treatment services and to make the integration of podoconiosis service in the government health system of Ethiopia more feasible.

Whether an individual was willing to pay for podoconiosis was strongly associated with the financial status of the household. Furthermore, individuals who were not willing to pay were members of households with half as much monthly income as compared to those that were willing to pay. Previous studies have shown that financial limitation was the primary barrier against consistent use of footwear among podoconiosis patients [7,8]. The study participants in our study appeared to have lower socioeconomic status because the majority of the respondents (64.1%) perceived that they were poorer than the village average, and half of the study participants were barefoot during the interview. Moreover, approximately twenty percent of the study participants reported at least one additional podoconiosis-affected household member, which can lower the economic productivity of households due to the morbidity from acute adenolymphangitis (ALA: hot, tender, painful symptoms in the lower legs) experienced by affected individuals as reported by previous studies in northern Ethiopia [19,20].

Several factors influenced WTP of podoconiosis patients. Factors that were associated with increased WTP can be generalized under four categories: (i) higher socio-economic status; (ii) older age, (iii) female sex, and (iv) more awareness about podoconiosis and modern health care. First, the positive association between higher socio-economic status and WTP in our study is supported by several other studies, showing that people’s WTP behavior parallels their ability to pay [21]. Similar findings have been reported in previous studies conducted in Ethiopia on WTP for injectable contraceptives [16] and insecticide-treated bed nets [17]. Second, older podoconiosis patients had greater WTP. This may be because the onset of podoconiosis is usually in the third decade of life and the disease advances with age urging patients to seek health care due to multiple episodes of ALA, limitations of physical activity, morbidity, and pain [19,20,22-24]. In addition, a study in southern Ethiopia has shown that because of financial resource barriers in the household, shoes (the main components of podoconiosis treatment and prevention) are often purchased for older household members first rather than for children [25]. Third, our finding of WTP by more women as compared to men may be because more women experience ALA including more frequent episodes and more bed days per episode [23]. In the present study, more women than men were barefoot during the interview (55% vs. 45.5%) and more women had never worn shoes (38.1% vs. 31.8%) consistent with previous studies showing that women possessed fewer means to prevent podoconiosis compared to men [6,14], resulting in a higher demand for treatment service. Moreover, stigmatization and poor marriage prospects for podoconiosis affected women [5,8] may be additional social factors that motivate women to be willing to pay more for treatment than men. Fourth, factors such as the possession of a functional radio and previous experience of treatment seeking for other illnesses could enhance awareness about modern health care and this has also been found to be associated with increased odds of WTP for insecticide-treated bed nets [17].

The study participants’ median WTP based on the open-ended question was approximately twenty percent of IOCC’s total treatment cost and a quarter of IOCC’s shoe production cost. The WTP amount declined only slightly until after the total cost was 15% of IOCC’s total treatment cost. These findings show new opportunities to introduce cost-recovery strategies in podoconiosis treatment programs. The price that is both affordable by most podoconiosis-affected households and optimal for cost-recovery should be assessed in program planning.

We acknowledge that there are some limitations to this study. The sample was selected from the treatment program waiting list which may have biased results. For example study participants prior knowledge of IOCC’s highly subsidized podoconiosis treatment program services may have made them less willing to pay which would have underestimated the WTP amount. An additional limitation could be related to the representativeness of the results. WTP could vary by regional cultures and seasonally, so more research is needed to explore WTP which could be used in the national NTD programming and policy planning. The findings of this study needs to be validated using qualitative study methods, in socio-culturally and economically diverse areas in Ethiopia, and at different seasons of the year because individuals’ WTP varies with season as the majority of the study participants are farmers. Moreover, we have not assessed the test-retest reliability of the CVM due to resource and time constraints.

This study can serve as a foundation for future studies to set a price threshold for podoconiosis treatment services in both governmental and non-governmental facilities. For podoconiosis affected households that are not able to afford and not willing to pay, selective fee waiver certification could be introduced. A fee-waiver and exemption system is currently institutionalized in Ethiopia’s government health service institutions for essential health care services [26], and this can be adapted for podoconiosis treatment.

## Conclusions

This study showed a significant potential for introducing a cost-recovery system that could increase podoconiosis treatment service utilization and reduce the backlog of patients in the waiting list of IOCC due to inadequacy of resources. In Ethiopia, less than four percent of estimated podoconiosis patients have access to treatment [11]. Therefore, the promotion of self-financing could enhance the sustainability of treatment in podoconiosis programs or integrated initiatives such as morbidity management with filarial lymphedema. A subsidized cost-recovery scheme could reduced treatment costs and more feasibly integrate podoconiosis treatment services with other NTDs and the government’s primary health care system.

## Abbreviations

ALA: adenolymphangitis; CVM: Contingent valuation method; IOCC: International Orthodox Christian Charities; NTD: Neglected tropical disease; OR: Odds ratio; s.d.: Standard deviation; WTP: Willingness to pay.

## Competing interests

The authors declare that they have no competing interests.

## Authors’ contributions

FTA conceived the study. AT, FTA, MG, ST, and MW designed the study. AT supervised the fieldwork. AT, GT, and FTA analyzed the study. AT and FTA drafted the manuscript, and all authors interpreted the findings, and critically reviewed the manuscript for substantial intellectual content. All authors approved the final version of the manuscript.

## Pre-publication history

The pre-publication history for this paper can be accessed here:

http://www.biomedcentral.com/1471-2458/14/259/prepub
